# Foreign

**Published:** 1840-12-26

**Authors:** 


### FOREIGN.

*Medical Experiments.* By Dr. J. C. G. Jörg.

(Continued from p. 820.)

*Castor.*—The results of the experiments with this substance may be summed up in a few words; it has no effect but that of causing eructations. The highest dose taken was xxiv. grains; but Alexander, who, more than half a century ago, took it in much larger doses, came to the same conclusion. It seemed one day to raise a thermometer placed upon the pit of his stomach one degree.

*Musk.*—The doses of this substance varied from half a grain to fifteen grains. It excited eructation, oppression of the stomach, want of appetite, (sometimes increase of hunger,) and dryness in the œsophagus; confusion, vertigo, weight, and heavy pains in the head; it acts as a stimulant upon the intestinal canal, but more especially upon the brain. As the secondary effects of this stimulating power, we have yawning, sleepiness, prolonged and deeper sleep, and relaxation of the whole body. When musk excites the whole nervous system considerably (as it is wont to do in sensitive persons) its power extends to the muscles also, causing trembling and shaking, or, in larger doses, convulsions. Musk also increases the activity of the vascular system, making the pulse quicker and fuller, as appears from many of the experiments. Hence it belongs to the general stimulants, or remedies which heighten the *vis vitæ*; but on account of its especial action on the brain, which is perhaps reinforced by the continual odour from the stomach, it must be used with caution.

Dr. Jörg is of opinion that musk stands below camphor as a stimulant, and is especially inferior to it in cases where the organs of assimilation are much weakened. Unless, therefore, we wish particularly to profit by the narcotic power of musk, we shall in general do well to choose some other stimulant; partly, he says, because the smell of musk generally depresses the spirits of the patient and his friends, as it is almost universally looked upon as the last medicine prescribed before death; and partly because it is very dear and often adulterated.

The quantity of musk required for an effective dose was very different with different experimenters. Three grains with one did more than ten or fifteen with another, because the smaller dose was taken by very sensitive persons. But since this remedy, if prescribed in conformity with the rule *contrarium contrario*, will be taken only by those whose nervous sensibility is dulled, not sharpened, it is clear that the smaller doses which act upon the healthy would be inefficacious with the sick.*

Hence when the patient, anteriorly to his malady, has been very sensitive, from three to five grains will suffice for a dose; if rather torpid, from six to ten grains, or more, must be given. The interval between the doses should be eight or twelve hours.

*St. Ignatius's Bean.*—This remedy was first

* This rule, though just, seems to us to have been hitherto but little attended to by Dr. Jorg in his estimation of doses.—Translator's Note.

taken in the form of a tincture made with one part of the drug to eight parts of spirit. The doses varied from 4 to 200 drops, and met with very various powers of resistance in the club of experimenters. Thus Lippert, who seems to have been strychnine-proof, obtained only one half-fluid stool from 180 drops, and no effect at all from 200; while Meurer, in consequence of a dose of 40 drops, was attacked with the following symptoms: extreme giddiness, so that he could hardly stand upright; shooting pain in the head; tinnitus aurium; apparent motion of the surrounding objects: an incapacity of retaining the same idea for a moment; nausea; increased flow of saliva; and want of appetite. He slept better than he had done the preceding night after a dose of 32 drops, but he was attacked by headach at intervals for about 48 hours.

The society afterwards took the drug in powder rubbed up with sugar of milk, but the largest dose was only four grains.

Dr. Jörg informs us that the Faba St. Ignatii primarily sharpens the activity of the intestinal system and the brain, and, in a general point of view, stimulates both organs, but with many peculiarities. Among these are its remarkable influence upon the salivary glands, and, doubtless, upon the pancreas and the mesenteric glands; and also the disappearance and re-appearance of its effects. This, however, is not always the case; the symptoms caused by this drug do not always suffer a remission and an exacerbation, nor does the re-appearance observe any regular interval.

Dr. Jörg recommends its use in weakness of the stomach, and in weakness of the eyes connected with inactivity of the brain, as an alterative in chronic diseases, and as the means of breaking, by a sudden shock, a morbid chain of symptoms whether in mind or body.

Dr. Jörg informs us that the dose should usually be half a grain or a grain, but if the tincture be used, the dose should be somewhat more than proportionably greater. It should not be repeated till after an interval of 24 hours; nay, the interval may sometimes be prolonged to 48 or 72.

*Assafœtida.*—The doses which were taken of this drug varied from half a grain to fifteen grains. It appeared from the results that assafœtida is a powerful stimulant to the alimentary canal, from the mouth to the anus; but it stimulates the upper part of the tube, the œsophagus, the stomach, and the small intestines, more than the lower part, or large intestines. This is a natural consequence of its affecting the intestinal canal chiefly by a specific acridity, and this acridity being decomposed principally in the upper part of the tube. Hence assafœtida is a good remedy for assisting digestion, but not for opening the bowels. It likewise stimulates the brain, but it is probable that this is only a secondary effect, and is a result of its stimulating the abdominal ganglia, and increasing their sensibility. It likewise stimulates the circulation, the urinary and the genital organs.

From these properties, Dr. Jörg deduces that assafœtida is very erroneously prescribed in hysteria, and in many hypochondriacal cases. Nor is it prescribed only in unsuitable cases, but in over-large doses. Half a grain will in general be a sufficient dose, though there are cases where two, three, four, or five grains may be taken at once; the remedy must not be repeated oftener than every twenty-four hours at the most.

*Opium.*—The society first took this drug in the form of tincture prepared according to the Saxon Pharmacopœia, excepting that simple distilled water was substituted for cinnamon water. It was made by digesting one part of purified opium with three parts of rectified spirits and three parts of distilled water, until the opium was dissolved, and then filtering; the tincture, therefore, was about three times the strength of the London one. The doses varied from half a drop to thirty-six drops. When opium was taken in substance, the range of doses was from one-twelfth of a grain to three grains.

The following are the conclusions at which Dr. Jörg arrives:—

" Opium first of all attacks the brain, and that more than any other organ, causing rapid and considerable congestion in it; and hence, in the beginning, it causes, in a proper dose, the feelings of lightness in the head, (it seems as if the head itself was borne up by the air, as if it flew,) unusual cheerfulness, and also a state like intoxication; afterwards, confusion, giddiness, heaviness, oppression and pain in the head; lastly, sleepiness and even deep and sound sleep. Inasmuch as opium, like all the more powerful narcotics, chiefly attacks the anterior part of the brain, in so far does it especially affect the eyes and the nose, creating a sense of dryness there, and also secondarily obscures the sight, probably because it causes congestion in these organs.

" The stimulating power of opium, however, extends also to the whole nervous system; but the state of primary irritation frequently lasts so short a time, that it escapes our observation; for when the congestion in the brain has reached a considerable extent, (and, when opium has been taken, this often happens in a very short time,—in a few minutes, if the dose has been considerable,) the secondary state, or state of depression, comes on, particularly in the nerves under the influence of the will; and relaxation, weariness, faintness, immobility of the limbs, &c. supervene so rapidly, that in general we entirely overlook the preceding excitement. But if, on the other hand, opium is given in the proper dose, so that the primary excitation of the brain and nervous system is but moderate, those phenomena are offered to our observation which spring from an exaltation of nervous life; among these I number an increase of common sensation, or of the consciousness of existence, a more intense and exquisite perception of the external world, and greater activity in the muscles. But whether this state, with the corporeal and mental sensations flowing from it, lasts a short or long time, it is followed by the opposite condition, the intensity of which is proportioned to that of the previous excitement; with this peculiarity, however, that the depression lasts much longer than the preceding increase of nervous life.

"Next to the brain and nerves, opium exerts its stimulus especially on the intestinal canal, and primarily causes powerful contractions of the stomach and of the small intestines, and, in a less degree, of the large ones; hence it causes oppression of the stomach, and perceptible but not painful movements of the intestines, and griping and colic pains, with a desire of evacuating the rectum; and also debility, nausea, and lessened or increased appetite. But in this great organ likewise the primary excitement is followed by a secondary depression of the *vis vitæ*, and hence there come on constipation, retention of flatus, and a tympanitic state of the abdomen, with a frequent desire to go to stool, although for a long time the fæces cannot be evacuated. For a long time the contractions of the intestinal canal seem to remain too weak to move on the harder excrements.

"From the stimulating effects of opium upon the nervous system and the intestinal canal, we learn its cardinal operations, on which its other numerous effects depend. When given in the smaller doses it does not act upon the whole of the two symptoms just named, while in the larger ones it extends to the vascular system, the skin, the urinary organs, and also the genitals; in such a manner that it calls forth in them alterations of more or less importance and variety, according as it is used in larger or smaller doses, and finds in the body a greater or less susceptibility to its impressions. What morbid symptom cannot a remedy produce, which primarily and secondarily changes the condition of the whole nervous system, and of the intestinal canal, in the way that opium does? Can we be surprised if occasionally palpitation, quickening of the circulation, hardness, largeness, and fulness of the pulse, or retardation, smallness, and contraction of the beat, and even an intermission of several beats should arise; if the temperature of the body sometimes is increased and sometimes diminished; if at one time sweat breaks forth, at another the hot skin remains dry: if the urine deviated from the standard in quality or quantity; if the generative organs sometimes seem to be entirely quiescent, or sometimes too active; if rheumatic pains harass the extremities, &c. &c.? Nay, if in a more intense degree of its action, convulsions and apoplexy make their appearance?" (pp. 437, 439.)

Dr. Jörg comes to the extraordinary conclusion, that opium ought very seldom to be used. He tells us that for the last ten years he has very rarely used opium, and that he is firmly convinced that it is really indicated in very few cases of disease, but that in these few cases it is an indispensable medicine. The only ones he mentions are an excited, but not inflamed state of the intestinal canal, with increased secretion from its inner surface, and consecutive vomiting or diarrhœa; and an excessive secretion from the kidneys, the genitals, and the skin.

Dr. Jörg recommends that the dose of opium should not exceed from one-twelfth to one-fourth of a grain, at intervals of six, twelve, twenty-four, or more hours. When given as a clyster, in gruel or decoction of linseed, to soothe irritability of the bowels, the dose is to be from half a grain to a grain.

*Digitalis.*—A quarter of a grain was the smallest, and three grains the largest dose taken by any of the club, with one exception: Lippert carried the dose as far as ten grains, without any effect, though his medicine was weighed out from the vial which supplied the other members. (p. 445.) A few of the experimenters likewise took the tincture, infusion, or decoction.

The results of the experiments showed that digitalis acts primarily as a stimulus to the brain, the intestinal canal, the urinary and the genital organs; and, secondarily, as a sedative to the vascular system. We believe that the credit of having discovered that the sedative action of digitalis is preceded by a stimulant one, is entirely due to the Leipsic society.

It is due to the power which digitalis has of stimulating the brain, that Dr. Jörg attributes its want of success in hydrocephalus; in another of his works (*Ueber die Kinderkrankheiten*) he observes, that by means of his experiments he discovered in a few weeks what Gölis was sixteen years in finding out, namely, the inutility of digitalis in acute hydrocephalus.

Dr. Jörg likewise disapproves of the foxglove in all dropsies where there is decided inflammation, and in hooping cough; and doubts whether in many diseases of the heart its secondary or depressing power will not be unprofitable or injurious. Next to the powder, the decoction seemed strongest. The dose of the powder is not to exceed a quarter of a grain, half a grain, or, at most, a grain. The preparations being more or less uncertain, must be given in doses containing a proportionally larger quantity of the drug. The doses of foxglove, says he, are repeated too frequently; the intervals between them ought to be of twelve, twenty-four, or forty-eight hours.

*Tincture of Iodine.*—This preparation was made by dissolving forty-eight grains of iodine in an ounce of alcohol, and the doses varied from one to eighteen drops. The results showed that iodine acts primarily as a stimulus to the intestinal canal, from the mouth to the anus; and it would appear that it excites the parietes of the intestines in a manner similar to that of healthy and very concentrated saliva and pancreatic juice. Hence in healthy persons it produces a salt taste, increased secretion of saliva, increased hunger and thirst, perceptible and increased movements of the intestines, slight griping, discharge of flatulence, excrements, and so on. Its stimulating power, however, extends to the brain also, as is the case with all remedies which considerably quicken the activity of the intestinal canal; hence it causes confusion in the head and heavy pain, sometimes of one, sometimes of another part of it. It also augments the flow of blood to the trachea and lungs, bringing them into a state approximating to inflammation, or actually inflaming them. This action seems to extend even to the Schneiderian membrane, and hence it secondarily causes increased secretion of mucus in the bronchi and the cavities of the nose. As iodine so powerfully excites the intestinal canal, it must necessarily affect the urinary and genital organs when its action reaches a very high degree, or is kept up very long.

After a few more observations, Dr. Jörg concludes his account and his book by asking—

"Who would then wish to limit this splendid remedy merely to the treatment of bronchocele? We shall obtain most from it in diseases of the abdominal viscera, in weakness of the intestinal canal, in obstructions of the abdomen, in scrofula and similar maladies. Where particularly the vegetative processes of life are prostrate, and this morbid state is accompanied by diminished *vis vitæ*, it promises the greatest performances. Yet it must always be used with caution, lest it should excite inflammation, or too great a luxuriance of growth, or resolution of the tissues. (*Zu beträchtliche Wucherung oder Auflockerung.*) From two to eight drops every twenty-four or forty-eight hours will be the usual dose." (p. 500.) Abridged from Dr. Jörg's *Materialen zu einer Künftigen Heilmittellehre.* (Our scanty compendium can hardly give a notion of the German minuteness and diligence which are conspicuous in every page of the original. Its merits are undoubtedly very great. Yet, in many instances, Dr. Jörg appears to have concluded too hastily from the healthy to the sick. He believes in the discutient powers of arnica, though the society afforded no tumor to discuss; surely the anodyne and antispasmodic powers of opium are beyond the reach of doubt, though among the happy experimentalists of Leipsic there was not a pain to be soothed, nor a spasm to be resolved.—TRANSLATOR.)—*London Medical Gaz.*

---

*Experiments on the Motions and Sounds of the Heart, by the London Committees of the British Association for* 1838-39, *and* 1839-40.—The following report consists of two distinct portions, the former describing the experiments performed at King's College, in 1839, by the London Committee for 1838 and 1839, and the latter detailing those of the Committee for 1839-40, performed at the Marylebone Infirmary in the present year. The former series, performed in conjunction by Professor Todd, Dr. C. J. B. Williams, and the reporter, with occasional assistance from Dr. Roget, were successfully commenced but were not completed, owing to the difficulty of procuring subjects, and other circumstances beyond the control of the committee. No report of those experiments was, consequently, presented at the Birmingham meeting, or has yet been published; and an account is, therefore, now prefixed to the report of the proceedings of the committee for the current year.

The experiments of 1838-9 were performed with the view to determine the physical and pathological causes of certain modifications of the motions and sounds that are presented by diseases—a chief object being to ascertain how, by mechanical and other irritations, and by displacements of the heart, murmurs could be produced; how also by inflammation. Whether, for example, the pericarditic friction sounds depend on deficient lubrication of the pericardium, or vascular turgescence, or are dependent solely on the effusion of lymph;—how far, also, the natural sounds might be impaired by interrupting the action of the valves in the living subject, or by spontaneous or artificially excited abnormal action in the muscular parts of the cavities without structural lesion. Another inquiry was this—how far do the motions and sounds of the heart in the lower animals correspond with those of the human subject: whether, for example, in birds and other animals that differ more or less from man in their cardiac anatomy, there be not corresponding differences in the cardiac sounds and motions. To those questions the experiments for 1838-9 supply answers, in most cases, which are satisfactory, in the opinion of the committee, to as great an extent as could be calculated on from so limited a number of observations: they feel, however, that the experiments were too few to decide any point of much difficulty or importance, and that further trials, under more favourable circumstances, are very desirable.

The experiments referred to are the following:

*Experiments for* 1838-9.

OBS. I.—14th June. Present, Drs. Roget, Todd, Williams, and Clendinning.

*Subject*—An ass about three months old. Pulse about 60; regular. At 8 o'clock, A. M., a long fine needle, with a silver canula, was passed into the chest at the left margin of the sternum between the ribs to the depth of two inches. The needle immediately exhibited motions corresponding to those of the heart. The needle was withdrawn, and aqua ammoniæ, diluted with four or five parts of water, was injected through the canula. The pulsation of the heart became immediately weak and very irregular, with intermissions.

10 *o'clock.* Heart's action natural; pulse 77.

12 *o'clock.* Pulse 70. Occasionally irregularly accelerated for a few beats.

2 *P. M.* Still no abnormal sound.

5 *P. M.* Pulse 78.

15*th June*—7 A. M. Pulse about 80. At half past 7, half an ounce more of solution of ammonia was injected as before; after which pulsations weak and irregular at first, but afterwards regular. Pulse 96; strong, with clear sounds.

12 *o'clock.* Pulse 72. Sounds natural and regular. First sound somewhat prolonged, with suspicion of murmur.

16*th.* Sounds strong. Pulse 56. Canula again introduced at the root of the xiphoid cartilage into the pericardium. Some blood followed the needle; then some strong solution of salt was injected, whence irregular accelerated action of the heart.

4 *P. M.* No murmur present. Both sounds distinct. Intermission every fourth or fifth beat. (C.)

5 *P. M.* Pulse irregular. First sound double. Generally in triplets, followed by intermission, the second sound being absent in the weak strokes preceding the intermission, but distinct and loud at other times. Pulse 56, but variable. (W.)

17*th*—3 *P. M.* Pulse 56. Still occasionally retarded. Both sounds now rough. Roughness most apparent about the base of the left side, and scarcely audible in the carotids. (W. and T.)

18*th*—7 *A. M.* Dead. But yet warm. Much blood escaped from subclavian vein on opening chest, and coagulated afterwards. A mass of greenish-yellow lymph in the mediastinum. The cellular membrane highly vascular, and easily torn. Flakes of lymph on lower anterior left lung. External pericardium marked with many straight vessels, and intermediate red striæ, giving bright redness to the whole. Same, in a slight degree, the interior pericardium, which contained two ounces of yellow serum. At base of heart most redness. Cellular substance there somewhat infiltrated with serum. Whole interior of surface of heart healthy, except from slight thickening and opacity of mitral. A wound, plugged with lymph, found in anterior face of right ventricle.

Obs. II.—17*th.* *Subject*—An ass ten weeks old. Pulse 48. Regular and pretty strong. Animal weak. By pressing between the fingers and thumb the cardiac region, the thumb being on the third rib and left side, a loud blowing was excited with the first sound, which ceased on removing the pressure. After several repetitions of this experiment, a short filing sound heard (by two members of the committee) after the second sound, the first being clear. On repeating the pressure more strongly, two murmurs were heard, (by the same observers,) one with the first sound, and continuing after it, and one with the second sound, (which was also weakened,) and continuing after it.

After being fifteen minutes at liberty, the animal had a deep-toned blowing with first sound, which soon ceased, but the murmur after the second sound continued.

20*th*—8 *A. M.* Same murmur or filing after the second sound as before. A long needle was passed two and a half inches deep vertically to the fourth rib, along the upper margin three inches from the sternum. A strong double motion was given to the needle, and a blowing, resembling a cooing, accompanied the first sound. The heart's action was increased, though the animal seemed faint.

21*st.* Pulse 60. The needle again introduced three inches: as before the needle presented rhythmical movements, sternad and dorsad. That dorsad being slow and forcible, and synchronous with the first sound. That sternad being sudden, like a fall back from gravitation, and accompanying the second sound. A murmur of a blowing or whistling kind heard with the systole and diastole also, the latter variously described by different observers. Murmurs and sounds were variously altered and impaired by pressing the needle flat in different directions. On withdrawing the needle, murmurs were heard with systole and diastole, described as rasping and filing respectively. (W. and T.)

Natural sounds distinct. The needle was introduced a second and third time. After the third withdrawal of the needle a loud creaking was heard with both sounds by two observers, but no constant abnormal sound by the third. The creaking was reported (W. and T.) to continue some minutes, when the natural sounds returned, with only slight murmur with the second sound.

22*d.* Animal dead (7 A. M.) and cold. Considerable effusion of bloody serum in right pleura and mediastinum. Some ecchymoses and marks of perforation on left ventricle, with corresponding marks and changes on the pericardium. Perforation three-quarters of an inch below and behind, or nearer to the apex than the semilunar valves. The needle had transfixed the left ventricle, slightly wounding the mitral, and penetrating the posterior wall. The anterior lamina of the mitral had ecchymoses, and the posterior lamina was perforated near the edge, with a small fibrinous excrescence on valve. The wound passed through the opposite posterior wall of left ventricle, around which there was ecchymosis under the pericardium. The aortics were healthy.

Obs. III.—23*d.* *Subject.*—An ass of ten weeks old. Half-past 7 A. M. Pulse 60. Strong and distinct. A canula was introduced about an inch from the xiphoid cartilage, and for about an inch in depth, when a sound, first as of rubbing, afterwards as of blowing, accompanied the latter part of the systole. About an ounce of brine was then injected, when the pulsations became tumultuous and irregular, and the sounds obscure, with loud gurgling, (probably from injection of air.)

3 P. M.—Sounds obscure, but more distinct towards the base, when a short creaking (W. and T.) or blowing (C.) accompanied the first sound, which was not audible in the arteries. Pulse irregular.

24*th.*—3. P. M. Pulse 90, and regular. Sounds more distinct than yesterday, and towards the base of the heart accompanied by leather or parchment sound. Respiration laborious. Tender near the heart, but eats well, and is lively.

23*d.*—7 A. M. A loud parchment rubbing murmur with each of the sounds, which otherwise were distinct and natural. Pulse 80.

8 A. M.—Jugular vein opened. Copious hæmorrhage. Heart's action became rapid, with slight rubbing sound. Soon, however, became slow and strong, with superficial loud grating or rough sound, and, becoming gradually weaker, soon ceased.

One ounce of serum in left pleura. Two to three ounces in pericardium. External pericardium exhibited several striated patches of minute vessels. The cellular tissue was infiltrated with serum, and the serous membrane was easily detached. No lymph on the inner or free surface of the pericardium, but the heart was completely coated with thin, membraniform, soft lymp; thickest at the septum, and near the base. On the anterior and posterior surfaces numerous minute depressions or lacunæ were seen in the lymph. The lymph was easily removed. On the left ventricle near the apex was an oval space of an inch by an inch and a half of bright red patches, seeming partly vascular, partly ecchymotic, about the middle of which was a punctured wound, and a clot in the muscular tissue beneath, and some ecchymoses under the corresponding endocardium. The interior of the heart healthy. The serum from the pericardium after standing, separated into crassamentum and liquid.

Obs. IV.—23*d.* *Subject.*—A stout ass two months old. Pulse 60-70. Strong, with sounds very loud.

A quarter to 4 P. M. A needle was introduced at the upper edge of the fourth rib, three inches from the sternum, and one inch deep. The heart's action was accelerated, with obscure blowing with the systole. The needle being withdrawn, the heart's action was slower, with double creaking or leather sound reported by two observers as accompanying both sounds, which became stronger after a few minutes. Heart's action varying in regularity.

A quarter of an hour after. Leather sound at the site of the puncture; not at all at the apex. Natural sounds there quite distinct.

25*th.*—7 A. M. Both cardiac sounds loud, with sounds of friction at the basis cordis.

26*th.*—7 A. M. Normal sounds and friction sounds as before. A long needle three times introduced in different directions between the third and fourth ribs, and three to four inches from the sternum, without any marked effect, except sometimes, on strongly depressing the handle towards the sternum, a blowing with first sound was heard, the second sounds being normal. (Rubbing rather than blowing sound. C.)

On first introducing the needle, a scratching noise was sometimes heard, with the systole, as if from the point hitching against the heart's surface.

A fine curved tenaculum, about two inches in the curve, was then passed two to three inches from the sternum, behind the third rib, with the point towards the spine, and, when at the greatest depth, the handle was depressed towards the sternum, so as to move the hook outwards towards the ribs. A loud blowing then attended the first sound, which was still distinct. The second sound was wanting when the handle was most depressed, and obscure when the handle was somewhat raised, and restored to full force when the hook was withdrawn.

Half an hour after, the first sound was accompanied with blowing between the first and third ribs, while a friction sound accompanied the second sound. (The Reporter called it altogether friction sound, with both systole and diastole, but varying in hoarseness or roughness.) It was faintly audible in the carotids.

Half-past 3 P. M. Still slight friction and blowing (roughness only of frictions. Reporter,) increased after the animal struggled. The tenaculum was again introduced, and manipulated as before, and again the second sound was stopped by drawing at the root of the arteries, and restored on relaxing the hold, the first sound being accompanied by a loud whizzing, and the hoarse or rubbing sound being indistinct, if not absent.

On withdrawing the hook a transitory crackling was heard. On the introduction of the hook, the heart's action became tumultuous and irregular, and, on withdrawing it, very rapid. Pulse 112. Half an hour after the pulse still 112, and the first sound accompanied by murmur.

27*th.*—A quarter past 7 A. M. Sounds as before. Rough murmur, as of friction, with first sound especially. The animal then pithed, and artificial breathing established, and chest opened. Heart was acting vigorously, with the sounds distinct and normal.

*First Experiment.*

On introducing a finger into the right auriventricular orifice, first sound was accompanied with a whiz, and wanted its flapping beginning. The whiz was accompanied by a thrill sensible to the finger introduced. The whiz ceased, and the systolic flap returned on removing the finger.

This experiment was repeated several times with the like results.

*Second Experiment.*

The hook was introduced through the auricle to hook up the tendons of the mitral valves, when the flapping was impaired, not suppressed, and the whiz was uncertain.

*Third Experiment.*

A finger placed on auri-ventricular opening externally experienced the same vibratory or jerking motion as would be felt over the aortics; and to the eye the same motion was visible in the former during the first sound at its commencement, as at the arterial openings during the second sound. (C.)

*Fourth Experiment.*

A blunt bistoury was introduced into the auri-ventricular opening through the auricle, and the tendons of the septal lamina of the mitral were cut partially, when the flapping of the first sound was impaired, but not destroyed.

On examining the heart were found several marks of perforation of the large arteries, anteriorly to the valves; the perforations were just at the opening of coronary, but no valve was wounded. There were ecchymoses at the external mouths of the perforations, and attached to one wound was a clot with a fibrinous peduncle. On the surface of the right ventricle, corresponding to the infundibulum, the surface was injected, and roughened by lymph, with several scratches and punctures. The lymph was small in quantity and granular in appearance. A wound in the septum was plugged with lymph, as were all the flesh wounds in the interior of the heart.

Obs. V.—29*th.* *Subject.*—A donkey three months old. Half-past 7 A. M. Heart's action quite normal. A tenaculum passed four inches from the sternum, between the third and fourth ribs: the handle having been lowered towards the spine, there was a whizzing heard with the first sound; but the second sound was only a little weakened. The whiz or blowing continued after the experiment, with the systole, and after the flap of the valves, but soon became intermittent, and gradually disappeared.

30*th.*—A fine canula was passed through the sternum an inch from the xiphoid cartilage, and about 12 ounces of warm water were injected. The cardiac sounds became presently apparently distant, especially towards the sternum. On withdrawing the tube, the sounds were still distant, with little impulse, but were otherwise normal, except that occasionally the systole was accompanied by blowing during embarrassed respiration. A tumor formed under the integuments of the sternum, through which the cardiac sounds were very faintly heard, and without impulse. Heart's action much accelerated.

*July 4th.*—Animal pithed, and artificial breathing established. The experiments on the mitral valves then repeated. The left auricle was inverted by the finger, and the valves impeded or kept asunder by the finger in the auri-ventrical opening, when various murmurs accompanied or followed the first sound; the second sound being simply either much weakened or suppressed, and the normal sounds returned on the withdrawal of the finger. This experiment was often repeated with similar results.

A finger being placed on the exterior circumference of the mitral and aortic valves respectively at the same moment, similar jerking motions perceived in each at the closure of the valves and evolution of the two cardiac sounds. The finger when in the auri-ventricular opening was sensible of something like flapping, pushing, and stretching as it were, in and by the valves: and the supposed edge of the valves was felt tense in systole, and if divided by the point of the finger, the edges of the opposite valves were thought to give a feeling of resistance, such as valvular tension must cause, supposing such tension to occur. The first sound was protracted and dull, wanting the sharply defined beginning, such as a flap would give, when the valvular action was interrupted by the finger.

The first sound was obscure, but audible on extraction of the heart, when the organ was irritated and contractive.

Obs. VI.—*July 3d.* *Subject*—A turtle, weight 150 lbs. No distinct pulsation could be heard externally. After decapitation and removal of the callipée, the heart was felt by one of the committee pulsating regularly, and two distinct sounds were heard (W.) with an interval between; the heart ceased beating too soon to allow of the other member of the committee (C.) making any satisfactory observation.

Obs. VII.—*Comparative Observation.*—The observations of the committee on the motions and sounds of the heart had been previously made almost exclusively on donkeys and dogs —animals whose cardiac structure and modes of action are generally known to agree very nearly with those of the human subject. It was therefore thought very desirable to extend their investigations more widely over the scale, as by such means, it was thought some useful generalization might be obtained, and the views of the committee be at the same time subjected to a new and interesting test, and if sound fully confirmed, but if defective, corrected; and in any event that their future conclusions would be based on a greater variety of facts, and a more comprehensive induction. The committee, therefore, made arrangements for the purpose of visiting the Zoological Gardens, and examining as many of the animals as could easily be approached by strangers, for the purposes of auscultation, &c. Before visiting the gardens the committee met at the Hunterian Museum, for the purpose of inspecting the preparations illustrative of the physiology of the heart that exist in that national collection, and were obligingly assisted in their search by Mr. Owen, the distinguished professor to the Royal College of Surgeons. With the aid of the anatomical data collected at the Royal College of Surgeons, the committee then entered at once on their examination of the living animals. Before stating any particulars of our observations, it is proper to say that in our examination of the wilder animals we were much indebted to Mr. Youatt, the distinguished veterinary surgeon of the establishment, without whose kind assistance it would have been out of our power even to have attempted any thing in several instances. Even with Mr. Youatt's aid we found it extremely difficult in many cases to make satisfactory observations, so that in but a portion of the subjects was it found practicable for the whole of the committee to verify results to their satisfaction.

The animals sufficiently examined by all are distinguished in the following enumeration:—

1. The ostrich.
2. The ourang-otang.
3. The leopard.
4. The seal.
5. The Balearic crane.
6. The Brahmin bull.
7. The common crane.
8. The puma.
9. The Indian antelope.

Other animals examined to the satisfaction of some members of the committee were

10. The elephant.
11. The dromedary.
12. The antelope.
13. The water buffalo.
14. The giraffe.
15. The lion.
16. The nylghau.
17. The Wapiti deer.
18. The hyæna.

In No. 1, (the ostrich) the pulse at the heart was very vigorous, and about 60 in the minute. The systolic or first sound was long and obtuse, and the second or diastolic sound was short and rather obtuse.

In No. 2, (the ourang-otang) the pulse was quick, and the cardiac sounds and rhythm like those of the heart of a child very exactly.

In No. 3, (the leopard) the pulse was 60, the first sound normal, but the second rather indistinct, as compared with the human standard.

In No. 4, (the seal) pulse not materally different from the human. First sound long and obtuse, second sound short and clear.

In No. 5, (the Balearic crane) pulse 130 to 140. Animal phthisical. First sound long and obtuse, second sound indistinct.

In No. 6, (the common crane) first sound short, and no second sound heard.

In No. 7, (the Brahmin bull) pulse 80. Animal phthisical. First sound long and obtuse, second sound indistinct.

In No. 8, (the puma) pulse 86. Animal sickly, probably phthisical. Grating murmur with the first sound.

In No. 9, (the Indian antelope) long obtuse first sound; short flapping second sound.

In No. 10, (the elephant) pulse 36; long and obtuse first sound, and relatively short and flapping second sound.

In No. 11, (the dromedary) pulse 48: long and obtuse first sound, short second sound.

In No. 12, (the antelope) the first sound longer and duller, the second sound shorter and sharper.

In No. 13, (the water buffalo) pulse 60. Blowing and murmur after the first sound; no second sound heard.

In No. 14, (the giraffe) pulse 50. Second sound sometimes double.

In No. 15, (the lion) first sound long and obtuse, second sound short and flapping.

In No. 16, (the nylghau) first sound normal, second sound indistinct.

In No. 17, (the Wapiti deer) pulse 60; first sound long and obtuse, second sound short and flapping.

In No. 18, (the hyæna) long obtuse first sound, short second sound.

Some other animals were attempted, but without success, viz., the dzagetai or wild ass, the rhinoceros, the cassowary, and some others. As a general observation, the committee may state, that wherever the second sound of the heart could be distinguished, the character of both sounds, and the rhythm of the heart's motions, appeared to correspond with those of the human heart; due allowance being made for differences of size in the animals, differences of temperament, and the circumstances of excitement or of disease under which many of the animals laboured when they were subjected to auscultation, &c.*

EXPERIMENTS FOR 1839–40.

In consequence of having been appointed to conduct the experiments on the motions and sounds of the heart for the current year, without being associated with any colleagues, I thought it desirable to avail myself of the assistance of such of my friends, including the other members of last year's committee, as could attend, and I accordingly requested the co-operation of a considerable number of gentlemen known to the public. Of those several were enabled to attend on numerous occasions, and one of them, Dr. Boyd, resident physician of the St. Marylebone Infirmary, on every occasion, so that every observation and

* The conclusions from the preceding experiments will be subjoined with those of the experiments that follow in the report for the current year.

experiment has been witnessed by one, or in most instances several, of the following gentlemen, to several of whom I am indebted for very important assistance:—

Professor C. J. B. Williams; George Gulliver, Esq., F.R.S.; John George Perry, Esq.; Dr. G. Hamilton Roe; Dr. George Burrows; Charles Cochrane, Esq.; Dr. Rutherford; Francis Kiernan, Esq., F.R.S.; J. Siddell, Esq.; T. K. Pritchard, Esq.; Francis Samwell, Esq.; Dr. Edwin Harrison; R. A. Stafford, Esq.; Benjamin Phillips, Esq., F.R.S.; Dr. Robert Boyd; and other gentlemen, private friends of the Reporter, and the last four named gentlemen his colleagues in the staff of the St. Marylebone Infirmary.

The experiments were performed in a convenient locality immediately adjoining the St. Marylebone Infirmary, and principally on donkey colts of a few months old. In the latter part of the series other animals, and especially dogs, were used, partly for economy, and in order that the limited pecuniary resources at my command might not be prematurely exhausted; and partly because certain experiments contemplated were expected to prove more easily and decisively practicable on the larger heart of the ass, than on any smaller, such as that of the dog; and that in any event it was desirable to extend the range of observation as far as practicable over the animal scale.

The mode of preparation was in all cases nearly the same. In almost every case sensibility was withdrawn as completely as was practicable, by one method or other. In donkeys, I availed myself of the stupefying property of the woorara poison, for a packet of which I had been indebted since 1838 to Sir B. C. Brodie. The woorara was brought into operation by injecting a couple of grains of it, partly dissolved, partly suspended in water, into the external jugular vein, as practised by Mr. Mayo in an experiment of Dr. Hope's, and the injection was usually followed in a very few minutes, by complete insensibility. In smaller animals prussic acid was used in several instances, and in a few the subject was stunned by a blow on the head. Artificial breathing was used in every warm-blooded subject, by means of a bellows and long flexible tube kept loose in the trachea; the chest was opened, nearly as directed by Galen (de admin. anat.) and as practised by former committees, and 5 or 6 ribs, at least, were separated from the sternum, and broken near the articulation, and bent back over the vertebræ. In every case, whether during the preparation or subsequent observation, all convenient means were used, as advised by Galen, to prevent or lessen hæmorrhage, in order to avoid, as much as possible, the anomalous modes of action attending extreme vascular depletion, and to prolong the opportunities of observation and experiment.

The observations about to be detailed consist partly of experiments in continuation of the inquiries of former committees, and partly of experiments conceived and performed with a view to decide several points in dispute amongst physiologists of authority, which were not investigated by those committees, and which seemed to me yet unsettled, and at the same time important enough to call for direct experimental investigation. The following are the principal of those undecided questions.

1. With respect to the rhythm of the motions of the auricles and ventricles, several living distinguished physiological writers appear to hold, that those cavities act in strict alternation with each other, and not continuously or in immediate succession, the auricles being first in systole and diastole, and the ventricular actions being last before the Rest, as described by Steno, Harvey, Lancisi, Haller, Senac, &c.; and by Hope, Williams, Carlile, Pennock, and Moore, and other distinguished living experimentalists.

2. With respect to the share in the circulation due to the auricular systole, it has been declared to be active, and of much importance, by Harvey, Senac, and others; while several living writers of great weight, adhering apparently to the views of Galen, Vesalius, &c., seem disposed to refuse to the auricles any very influential or positively important share in the cardiac operations; for examples I may cite Dr. Elliotson, Prof. Bouillaud, Dr. Hope, Sir B. C. Brodie, &c.

3. With respect to the shape and dimensions of the ventricles in systole, it was held by Galen, Vesalius, Harvey, &c., that the heart is shortened in diastole, and lengthened in systole; but the observations of Steno, Lower, Lancisi, Haller, and others, gave currency to opposite views. Of late, however, the ancient opinion has been revived; for example by Professor Burdach and Professor Bouillaud, as I understand their observations, and by Drs. Pennock and Moore, the latest experimentalists on the subject that I know of, except my friends and myself.

4. With respect to the præcordial impulse, the great majority of physiologists, adhering unqualifiedly to the ancient opinion, advocated by Hippocrates and Galen amongst the Greeks, and by Vesalius, Harvey, Lancisi, Senac, Haller, Hunter, &c., ascribe the cardiac pulsation to a blow or stroke (in the popular meaning of those words) given by the heart's apex in systole to the ribs; and refer the apparent inaction in the heart, between its pulsations, to the retreat of the organ during its diastole inwards, and away from the walls of the chest. But in opposition to this view may be cited the experiments of several recent observers, and the arguments of Mr. Carlile, of Dr. Hope (in his last edition), of Mr. Bryan, of Dr. Billing, &c. &c.

5. With respect to the diastole of the heart, it was held by Galen and Vesalius to include a strong force of *suction*, by which principally the venous current was forwarded and the auricles were emptied; and this power of inhalation or suction has been adopted by numerous living authorities; *e. g.* Professor Bouillaud, Dr. Hope, and Dr. Copland; and has even been extended to the auricular diastole, *e. g.* by Professor Alison and Dr. Elliotson. The exertion, however, of any such force, has been distinctly denied to the diastolic state by Harvey, Lower, Senac, &c.; and recently by Dr. Billing and Dr. Arnott, as physical absurdities, and the opinion appears, Dr. Joy remarks, to rest on no satisfactory experimental evidence whatsoever.

6. In addition to active pulsations observed in certain animals in the veins, (as in hares, rabbits, dogs, fowls, frogs, &c.) there have been noted by several experimentalists, of whom it is sufficient to name the great Haller, certain passive pulsations, viz., an abrubt diastole of the vein attending the first part of the heart's systole, or the auricular contraction, and an abrupt systole of the vein attending the first part of the heart's diastole, or the dilatation of the auricle; but the connexion between this venous regurgitation and the auricular systole has been doubted by several apparently, and even denied by Dr. Elliotson.

7. Reverting to the auricular function, the systole of the auricles has usually been regarded as unattended by any intrinsic sound. Dr. Hope denies that any such sound occurs, and on physical grounds seems to affirm that it is not possible; and Dr. Joy calls the auricular systole a "silent" act. (Library of Practical Medicine.) Six months probably, or more, however, before the London Commissioner for 1840 had even begun his experiments, Drs. Pennook and Moore had, unknown to him, and his friends, detected, as they conceived, an auricular systolic sound in a series of very interesting experiments, of which an account is published in the American Journal of Medical Science, No. 50, Feb. 1840.

8. The following, often agitated, and still moot points, have appeared to the reporter in like manner to stand in need of further examination, *e. g.* 1, the sizes of the cavities, &c., with respect to each other; 2, the production of sound by certain muscles while vigorously contracting; 3, the rhythm of the cardiac and arterial pulse; &c. &c. Finding on all the preceding points considerable difference of opinion, and perceiving that, in many instances, the decisions of highly distinguished and leading physiological writers were at variance with what he considered to be the best hitherto recorded experiments and observations, the Reporter found forced on his mind the conviction that on all or most of those points further data were wanting, and experiments less ambiguous, and more pointed and conclusive. Under such impressions the Reporter felt himself at liberty, if not positively called on, to advert to the various questions above alluded to, which had not been handled by former committees, provided that by any unlooked-for good fortune, if not through some new and happier experimental combinations, he should succeed in eliciting pertinent and decisive facts. Acting on such views, he has put to the test of experiment, to a greater or less extent, several of those questions, with results now to be stated.

It may be proper to mention that the instrument used in auscultation was exclusively the flexible ear tube; the wooden stethoscope, inconvenient in most cases, being found quite unsuited for such experiments.

Obs. 1.—*June 11th. Subject*—A donkey about ten weeks old, and sound in all respects. *Phenomena*—Various spontaneous irregularities in the cardiac action and sounds; jerking upwards, &c. of periphery of internal valves in systole; appearances of auricles in action; effect of valvular obstruction on first sound.

Heart, when exposed, acting strongly and quickly; second sound indistinct, and loud murmur with first sound.

*Section* 1.—On placing the finger on the outer periphery of the mitral valves, an upward jerk and thrilling motion sensible, similar to that observed over the arterial valves.

*Section* 2.—At the moment of auricular systole there was noted a dimpling of the appendix, and an abrupt contraction in all its dimensions, and a sinking, as it were, downwards and inwards, followed by a gradual return to the state of prominence and distension that characterize the auricular diastole. Several times was observed a slight and partial active contraction of the auricle, followed by relaxation in the intervals of full and complete auricular systole and diastole, as if from transient spasmodic action.

*Section* 3.—The second cardiac sound observed at intervals to be for many minutes together wholly wanting, without obvious cause, no operation upon the mitral or other valves having been hitherto attempted.

*Section* 4.—The left auricle was inverted successively by the finger, and by a probe, so as to impede the action of the mitral valves. The finger was sensible, when placed in the mitral orifice, of an abrupt though gentle concussion in the systole of the ventricle, and (it seemed to me) as if it were pushed by a cord or membrane stretched obliquely across the passage, and brought suddenly to a state of tension; and, at the same time, the sensation of jerking upwards was much less distinct when the finger was placed over the valve externally. The probe, also, when held loosely in the orifice (enveloped like the finger in the inverted appendix of the auricle) was felt and seen to be pushed back in each systole between the fingers.

*Section* 5.—At the moment of introducing the inverted appendix into the internal opening, the sharp well-defined beginning of the first or systolic sound was wanting or obscure; and that sound seemed to several observers less abrupt and more gradual than normal in its development.

Obs. II.—13*th*. *Subject*—A stout ass of two to three weeks old. *Phenomena*—Abnormal murmurs without structural defect. Motions of the ventricle in systole, as apparent to the eye and hand. Same of the auricles. Rhythm of the motions of auricles and ventricles. Auricular hæmorrhage not suspended in diastole, and augmented in systole of auricle.

*Section* 1.—Heart acting normally and vigorously before the injection of woorara. Immediately after the operation was completed a murmur was observed with the second sound, or diastolic sound with a slow cardiac action, the first or systolic sound being normal.

*Section* 2.—In systole motion first indistinctly observed at the fundus, especially on the right ventricle, where any phenomena about the arterial orifice are most easily observed in an animal lying on the right side; apex almost simultaneously moved with fundus. These systolic motions in the ventricles were preceded by a dimpling and shrinking inwards and downwards of the auricular appendixes, but by a very minute interval, so that the auricular motion seemed, as it were, but the first portion of a more extensive movement affecting the whole heart.

*Section* 3.—Before opening the pericardium, needles were passed horizontally through that organ without wounding the heart, and so that they lay exposed to the eye in their whole length, except the minute portion actually penetrating the pericardium, and over the following points, viz. over the auricle, over the periphery of the mitral orifice, and over the apex; and observation was made through a roll of paper employed to limit the field of vision, and a succession of motions was distinctly noted; first about the fundus and insertions of the great arteries, and most strikingly over the appendices auricularum, and thence propagated, as it were, towards the free extremity of the heart, being perceived in the body of the ventricles next after the fundus, and at the apex last, as if an impulse in a compressed fluid, or a wave, commencing about the insertion of the arteries, had been propagated along the heart from fundus to apex.

*Section* 4.—After opening the pericardium, small triangular bits of white card were applied so as to adhere to the left appendix, fundus of left ventricle, middle of same cavity, and close to the apex; and observation was made, as in last experiment, through rolled paper, and with like results.

*Section* 5.—And this seeming propagation of motion was perceived next in another way—viz. by pressing gently on the fundus and body of the ventricle, and on the apex, by which was elicited a sensation, or series of sensations, as of a progressive movement of an undulatory character, directed from fundus to apex, and resembling, to a considerable extent, that given by a dropsical abdomen, or a hydrocele, &c., when appropriately percussed.

*Section* 6.—During a very vigorous action of the auricles, and at a somewhat advanced period of the observation, the shrinking and dimpling in its centre of the appendix in auricular systole was likened by several observers to an effect either of suction, or of some traction exerted on the appendix from some point about the auriventricular opening, more especially because it seemed often separated in time from the ventricular tension, roundness, and impulse, (or systole,) by a scarcely perceptible interval.

*Section* 7.—Towards the end of the observation, and during a tolerably regular and vigorous action of the heart, the tip of the left auricle was snipt off, after which the contractions of the appendix became indistinct, those of the ventricle being at first little affected. On the instant of cutting off the appendix, a profuse flow of blood occurred, in a stream slightly increased by a jet during systole of the auricle, and continued without any jet during diastole.*

Obs. III.—22*d*. *Subject*—A donkey about six months old, in good health. *Phenomena*—Results of application of pressure in various ways to the ventricles. Rhythm and manner of motions of fundus and apex in systole and diastole. Motions in the arteries and over the valvular orifices. Action of sinuses in systole of auricles. Shortening of heart in systole. Effects of wounding an auricle, &c. &c.

*Section* 1.—Callipers were applied to the ventricles, as if to take the diameter of the heart. The legs of the callipers before use had been fastened together by an elastic cord of considerable resisting power. In whichever direction the instrument so prepared was made to embrace the ventricles, whether exactly transversely or obliquely, the uniform result was, that the legs of the instrument were separated with force, and receded from each other in each systole, and approached each other in each diastole, with depression of the part of the parietes they pressed on, which depression wholly disappeared in the systole, giving place to an opposite state of the parts, or to a state of convexity and even of protrusion in the central parts.

*Section* 2.—The finger and thumb were then applied to opposite sides of the ventricles, and

* The heart ceased to beat after about three-quarters of an hour of observation, owing to the inflating tube becoming obstructed by a clot of blood which escaped timely detection; it was quite healthy; the ventricles appeared not to differ in size.

were felt to be abruptly pushed outwards in systole, and to approach each other in diastole, if acted on even slightly by the flexor muscles, and with marked depression of the parietes in diastole, during which no sense of active resistance was experienced.

*Section* 3.—A wooden stethoscope was then placed on the ventricles, and kept erect by means of a roll of paper large enough to give the instrument full freedom of motion, and the uniform result was, that, wheresoever placed on the ventricles, the stethescope was heaved up with a jerk at each systole, (to the height of half an inch near the fundus,) and subsided at once in diastole, causing in the parietes a deep depression, which was wholly removed by the systole, and succeeded by an opposite shape of the surface.

*Section* 4.—To the eye and hand the fundus appeared to become round, hard, and elevated, and to give impulse somewhat sooner than the apex, as if the systole was developed earlier about the fundus than at the free extremity of the heart.

*Section* 5.—To the fingers, during the systole of the ventricles, a feeling was communicated, as of an undulation in a compressed fluid, very distinct, and directed from fundus to apex, and resembling sensations familiar to clinical physicians in ascites, hydrocele, &c., when properly percussed.

*Section* 6.—On touching the arteries close to the heart, a feeling as of efflux and reflux was very distinct, especially in the aorta; the former coinciding with the systole of the ventricles, the latter with the diastole. At the same moment with the outward current in the arteries, or during the ventricular systole, a peculiar jerking upwards of the periphery of the auriventricular orifices, and a similar eccentric movement was observed over the arterial openings during the reflux current or undulation in the vessels, or during ventricular diastole.

*Section* 7.—The sinuses of the auricles were found by the touch to contract vigorously, before the ventricles considerably, and even before the shrinking, &c., or systole of the appendices.

*Section* 8.—Small triangular pieces of white card were made to adhere to the fundus and apex cordis respectively, and observation was made through a roll of paper sufficiently large to take in at a convenient distance both extremities of the organ, and held so that each white object rested on a distinct limb of the tube's mouth, and every change of distance between the points dotted white was readily detectible; and the uniform result was, that the apex approached the base in systole, and receded again in diastole, and the mean range of oscillation seemed about one-third of an inch.

*Section* 9.—While the heart still acted, but with much diminished force, a cut was made in the right auricle, and a copious flow of blood obtained, having slight jets in the auricular systole, and immediately before the ventricular hardening, elevation, &c., but being continuous during diastole.

*Section* 10.—The ventricles appeared not to differ in size on careful examination post-mortem cordis.—*London Medical Gazette.*

(*To be continued.*)

---

*The Hydrostatic Test in cases of Infanticide.*—At a recent meeting of the Westminster Medical Society, Dr. Guy brought this subject before the notice of the Society. The fact to which he would allude he considered of value, as removing at least one of the objections which had been raised to the hydrostatic test. A short time ago Dr. Reid had presented him with a still-born child, which had been brought into the world, at the eighth month of pregnancy, by the induction of premature labour, which was rendered necessary, in consequence of the mother having a deformed pelvis. The child presented by the breech, and the pulsation in the cord had ceased long before the head was delivered by means of the forceps. In fact, there could be no question that the child was still born, and had not respired. Sixty-three hours after birth, Dr. Guy proceeded to examine the body. There was not the slightest trace of putrefaction, either externally or internally; the lungs were free from all trace of such a change. On looking at these organs, however, attentively, one or two vesicles, about the size of peas, were observed on the lower lobe of the right lung, and situated between the substance of the lung and the pleura. The appearance presented by these vesicles was precisely such as would indicate the presence of emphysema, if it could be supposed possible that emphysema *could* exist in lungs which had never been employed in respiration. He thought the presence of these vesicles sufficiently curious to entitle the lungs to a place in the museum of King's College; he therefore put them in a gally-pot, intending to take them to that institution. On looking at the lungs again, in two hours after, he was surprised to find that the whole of the lower lobe of the right lung was covered with these vesicles, which gave the appearance of minute mercurial injection. He was now convinced that this appearance depended on an incipient change of structure in the organs—in fact, on the first process of change which preceded the more ordinary signs of putrefaction. This fact explained those cases of supposed emphysema in still born children; which affection was considered by some authors to offer a very valid objection to the hydrostatic test. It was to be regretted that, by claiming too much for this test, it had lost some of its just reputation.

Many authors had pursued the subject rather as one of feeling than of science, and some had condemned the test with the anxiety of erring only on the side of humanity.—*Lancet.*

